# Small Lesions Evaluation Based on Unsupervised Cluster Analysis of Signal-Intensity Time Courses in Dynamic Breast MRI

**DOI:** 10.1155/2009/326924

**Published:** 2010-04-01

**Authors:** A. Meyer-Baese, T. Schlossbauer, O. Lange, A. Wismueller

**Affiliations:** ^1^Department of Electrical and Computer Engineering, Florida State University, Tallahassee, FL 32310, USA; ^2^Institute for Clinical Radiology, University of Munich, 81377 Munich, Germany; ^3^Department of Biomedical Engineering, University of Rochester, Rochester, NY 14642, USA

## Abstract

An application of an unsupervised neural network-based computer-aided diagnosis (CAD) system is reported for the detection and characterization of small indeterminate breast lesions, average size 1.1 mm, in dynamic contrast-enhanced MRI. This system enables the extraction of spatial and temporal features of dynamic MRI data and additionally provides a segmentation with regard to identification and regional subclassification of pathological breast tissue lesions. Lesions with an initial contrast enhancement ≥50% were selected with semiautomatic segmentation. This conventional segmentation analysis is based on the mean initial signal increase and postinitial course of all voxels included in the lesion. In this paper, we compare the conventional segmentation analysis with unsupervised classification for the evaluation of signal intensity time courses for the differential diagnosis of enhancing lesions in breast MRI. The results suggest that the computerized analysis system based on unsupervised clustering has the potential to increase the diagnostic accuracy of MRI mammography for small lesions and can be used as a basis for computer-aided diagnosis of breast cancer with MR mammography.

## 1. Introduction

**Figure 1:**
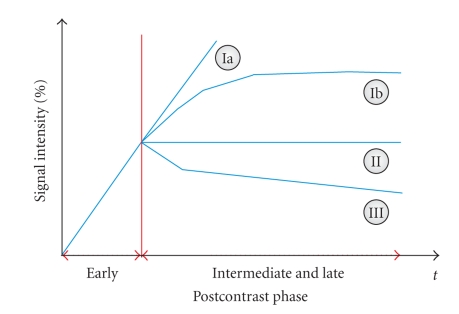
Schematic drawing of the time-signal intensity (SI) curve types [2]. Type I corresponds to a straight (Ia) or curved (Ib) line; enhancement continues over the entire dynamic study. Type II is a plateau curve with a sharp bend after the initial upstroke. Type III is a washout time course. The initial contrast enhancement *s* *a*_*i*_ is given by (4). In breast cancers, plateau or washout-time courses (type II or III) prevail. Steadily progressive signal intensity time courses (type I) are exhibited by benign enhancing lesions.

Breast cancer is one of the most common cancer among women. Dynamic magnetic resonance imaging (MRI) of the breast was reported to be a highly sensitive method for detection and further evaluation of clinically, mammographically, and sonographically occult cancers [1]. However, the limited specificity of breast MR imaging continues to be problematic. Two different approaches are mentioned in literature [2] aiming to improve the specificity: (1) single-breast imaging protocols with high spatial resolution offer a meticulous analysis of the lesion's structure and internal architecture and are able to distinguish between benign and malignant lesions and (2) lesion differential diagnosis in dynamic protocols is based on the assumption that benign and malignant lesions exhibit different enhancement kinetics. In [2], it was shown that the shape of the time-signal intensity curve represents an important criterion in differentiating benign and malignant enhancing lesions in dynamic breast MR imaging. The results indicate that the enhancement kinetics, as represented by the time-signal intensity curves visualized in Figure 1, differ significantly for benign and malignant enhancing lesions and thus represent a basis for differential diagnosis. In breast cancers, plateau or washout-time courses (type II or III) prevail. Steadily progressive signal intensity time courses (type I) are exhibited by benign enhancing lesions. Also, these enhancement kinetics are shared not only by benign tumors but also by fibrocystic changes [2].

The success of CAD in conventional X-ray mammography motivated the research of automated diagnosis techniques in breast MRI to expedite diagnostic and screening activities.

 A standard multilayer perceptron (MLP) was applied to the classification of signal-time curves from dynamic breast MRI in [3]. Breast MR segmentation and lesion detection is accomplished based on cellular neural networks in [4] and a 100% detection sensitivity is reported. In [5], the performance of a backpropagation neural network based on kinetic, morphologic, and combined MR features was shown to be comparable to that of an expert radiologist. The same type of neural networks was used for breast MRI lesion classification in [6]. As inputs, a subset of 13 features out of a total of 42 features describing lesion shape, texture, and enhancement kinetics was selected. The main result was that the performance of the human readers significantly improved when aided by a CAD system. It could be shown that specificity at a sensitivity of 90% was 0.505 for lesion classification without CAD assistance and 0.807 for classification with CAD assistance. Mean shift clustering in connection with automated selection of the most suspicious cluster resulted in accurate ROIs in breast MRI lesions, as shown in [7]. In [8], a fuzzy c-means clustering-based technique was tested for automatically identifying characteristic kinetic from breast lesions. By using four features extracted from these curves (maximum contrast enhancement, time to peak, uptake rate, and washout rate of the lesion kinetics), it was demonstrated that the prototype curves determined by the fuzzy classifier outperform those determined based on averaging over an ROI determined by an experienced radiologist. Three different classifiers, a multilayer perceptron, a threshold classifier, and a nearest-neighbor classifier, were employed for the classification of signal-time curves in [9]. As feature vectors, both the complete signal-time curve and select descriptive parameters derived from these curves were used. It was shown that the quantitative classifiers can support the radiologist in the diagnosis of breast lesions.

 A major disadvantage of most supervised techniques is the fixed number of input nodes which imposes the constraint of a fixed imaging protocol, as shown in [10]. Delayed administration of the contrast agent or a different temporal resolution has a negative effect on the classification and segmentation capabilities. Thus, a change in the MR imaging protocol requires a new training of the CAD system. A signal time-series is obtained for every pixel and has to be interpreted given the experimental conditions. Thus, supervised and unsupervised represent two different techniques when it comes to biomedical signal analysis. The supervised technique represents a model-driven approach that tries to interpret time-series as a result of dynamically changing input parameters, given a specific model that defines the pattern of interaction between the underlying physiological processes and the observed signal dynamics. However, this model-based, supervised technique is based on the underlying knowledge of experimental conditions and model assumptions. This aspect makes it difficult or impossible and therefore* unsupervised techniques represent better training approaches* for expert advanced CAD systems since they fit the model to the observations and analyze and visualize the data in an exploratory manner.

 An important aspect remains the fact that many of these techniques were applied on a database of predominantly tumors of a size larger than 2 cm. In these cases, MRI reaches a very high sensitivity in the detection of invasive breast cancer due to both morphological criteria as well as characteristic time-signal intensity curves. However, the value of dynamic MRI and of automatic identification and classification of characteristic kinetic curves is not well established in small lesions when clinical findings, mammography, and ultrasound are unclear.

 In the present study, we design and evaluate an unsupervised CAD system for the diagnosis of* small breast masses* with a diameter of only a few mm. For those lesions containing only a small number of voxels, morphologic criteria can hardly be evaluated. To overcome the above mentioned problems, we employ a “neural-gas” network as a quantization method that focuses strictly on the observed complete MRI signal time-series, and enables a self-organized data-driven segmentation of dynamic contrast-enhanced breast MRI time-series w.r.t. fine-grained differences of signal amplitude, and dynamics, such as focal enhancement in patients with indeterminate breast lesions. This method is developed, tested, and evaluated for functional and structural segmentation, visualization, and classification of dynamic contrast-enhanced breast MRI data. In addition, a comparison with another unsupervised method, the minimal free-energy vector quantization, and the conventional segmentation method is performed. We will show that the inspection of the clustering results represents a unique practical tool for the radiologists enabling a fast scan of the data set for regional differences or abnormalities of contrast-agent uptake. The proposed technique contributes to the diagnosis of indeterminate breast lesions by non-invasive imaging.

## 2. Material and Methods

### 2.1. Patients

A total of 40 patients, all female and age range 48–61, with indeterminate small mammographic breast lesions were examined. All patients were consecutively selected after clinical examinations, mammography in standard projections (craniocaudal and oblique mediolateral projections), and ultrasound. Only lesions BIRADS 3 and 4 were selected where at least one of the following criteria was present: nonpalpable lesion, previous surgery with intense scarring, or location difficult for biopsy (close to chest wall). All patients had histopathologically confirmed diagnosis from needle aspiration/excision biopsy and surgical removal. Breast cancer was diagnosed in 31 out of the total 40 cases. The size of malignant tumors ranged from 0.5 mm to 2.0 mm, average size was 1.06 mm, the size of benign tumors ranged from 0.8 mm to 1.8 mm, and average size was 1.2 mm.

### 2.2. MR Imaging

MRI was performed with a 1.5 T system (Magnetom Vision, Siemens, Erlangen, Germany) equipped with a dedicated surface coil to enable simultaneous imaging of both breasts. The patients were placed in a prone position. First, transversal images were acquired with an STIR (short TI inversion recovery) sequence (TR = 5600 ms, TE = 60 ms, FA = 90°, IT = 150 ms, matrix size 256 × 256 pixels, slice thickness 4 mm). Then a dynamic T1 weighted gradient echo sequence (3D fast low angle shot sequence) was performed (TR = 12 ms, TE = 5 ms, FA = 25°) in transversal slice orientation with a matrix size of 256 × 256 pixels and an effective slice thickness of 4 mm.

 The dynamic study consisted of 6 measurements with an interval of 83 seconds. The first frame was acquired before injection of paramagnetic contrast agent (gadopentatate dimeglumine, 0.1 mmol/kg body weight, Magnevist, Schering, Berlin, Germany) immediately followed by the 5 other measurements. The initial localization of suspicious breast lesions was performed by computing difference images, that is, subtracting the image data of the first from the fourth acquisition. As a preprocessing step to clustering, each raw gray level time-series *S*(*τ*), *τ* ∈ {1,…, 6} was transformed into a signal time-series of relative signal enhancement *x*(*τ*) for each voxel, the precontrast scan at *τ* = 1 serving as reference. Thus, we ensure that the proposed method is less sensitive to changing between different MR scanners and/or protocols.

### 2.3. Computer-Aided Diagnosis (CAD) System

**Figure 2:**
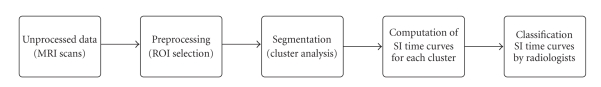
Diagram of the CAD system employed for the small mammographic lesion evaluation: the six MRI scans for each subject undergo a signal preprocessing, the ROI is determined and all voxels within it are subject to cluster analysis. The obtained time-signal intensity (SI) curves are compared to the four Kuhl classes from Figure 1 and automatically assigned to a class by the supervised neural network.

The small lesion evaluation is performed by an automated computer-aided diagnosis system based on preprocessing of the signal-intensity time-courses, segmentation of signal-intensity time-courses, and then automated evaluation of the time-signal intensity curve based on an unsupervised classifier. The flow diagram of the CAD system is shown in Figure 2.

In the present study, we selected only lesions with an initial contrast enhancement ≥50% for a comparative analysis between the conventional segmentation analysis method and the unsupervised classifier.

### 2.4. Data Clustering

The employed classifier—the “neural-gas” network—is based on grouping image voxels together based on the similarity of their intensity profile in time (i.e., their time courses). Another important feature of the presented algorithm compared to SOM is that it does not require a prespecified graph (network). In addition, it can produce topologically preserving maps, which is only possible if the topological structure of the graph matches the topological structure of the data manifold. In cases, however, where it is not possible to a priori determine an appropriate graph, for example, in cases where the topological structure of the data manifold is not known a priori or is too complicated to be specified, Kohonen's algorithm necessarily fails in providing perfectly topology preserving maps.

Let *n* denote the number of subsequent scans in a dynamic contrast-enhanced breast MRI study, and let *K* be the number of voxels in each scan. The dynamics of each voxel *μ* ∈ {1,…, *K*}, that is, the sequence of signal values {**x** ^*μ*^(1),…, **x** ^*μ*^(*n*)} can be interpreted as a vector **x** ^*μ*^(*i*) ∈ **R** ^*n*^ in the *n*-dimensional feature space of possible signal time-series at each voxel.

Cluster analysis groups image voxels together based on the similarity of their intensity profile in time. In the clustering process, a time course with *n* points is represented by one point in an *n*-dimensional Euclidean space which is subsequently partitioned into clusters based on the proximity of the input data. These groups or clusters are represented by prototypical time-series called codebook vectors (CVs) located at the center of the corresponding clusters. The CVs represent prototypical time-signal intensity curves sharing similar temporal characteristics.

Vector quantization (VQ) represents a fast clustering technique for feature vectors describing pixel time courses in breast MRI. VQ approaches determine the cluster centers **w**_*i*_ by an iterative adaptive update based on the following equation [11]:

(1) $$w_{i}\left(t+1\right)=w_{i}\left(t\right)+\alpha \left(t\right)a_{i}\left(x\left(t\right),C\left(t\right),\kappa \right)\left(x\left(t\right)-w_{i}\left(t\right)\right),$$

where *α*(*t*) represents the learning parameter, *a*_*i*_ a codebook *C*(*t*) dependent cooperativity function, *κ* a cooperativity parameter, and **x** a randomly chosen feature vector.

#### 2.4.1. “Neural Gas” Network

The “neural-gas” algorithm [12] is an efficient clustering approach which, applied to the task of vector quantization, (1) converges quickly to low distortion errors, (2) reaches a distortion error [12],(which measures the fidelity of data encoding and is given by the squared Euclidean distance between the data vectors and the corresponding approximating reference vectors)lower than that from self-organizing map (SOM), and (3) at the same time obeys a gradient descent on an energy surface representing the error function of the learning algorithm.

Instead of using the distance ||**x** − **w**_*i*_|| or of using the arrangement of the ||**w**_*i*_|| within an external lattice, it utilizes a neighborhood-ranking of the reference vectors **w**_*i*_ for the given data vector **x**.

The learning rule for the “neural-gas” network is [12]

(2) $$w_{i}\left(t+1\right)=w_{i}\left(t\right)+\alpha \left(t\right)\exp\;\left\{-k_{i}\left(x,\frac{w_{i}}{\lambda }\right)\right\}\left(x\left(t\right)-w_{i}\left(t\right)\right),$$

where *k*_*i*_ = 0,…, *N* − 1 represents the rank index describing the “neighborhood-ranking” of the reference vectors **w**_*i*_ to the data vector **x** in a decreasing order, *N* is the number of units in the network, and *λ* determines the number of neural units changing their synapses with every iteration. The step size *α* ∈ [0,1] describes the overall extent of the modification.

It was shown by Martinetz et al. [12] that the average change of the reference vectors corresponds to an overdamped motion of particles in a potential that is given by the negative data point density. Superimposed on the gradient of this potential is a “force”, which points toward the direction of the space where the particle density is low. This “force” is the result of a repulsive coupling between the particles (reference vectors). In its form it resembles an entropic force and tends to homogeneously distribute the particles (reference vectors) over the input space, like the case of a diffusing gas. This suggests the name for the “neural-gas” algorithm. It is interesting also to mention that the reference vectors **w**_*i*_ change their locations slowly but permanently, and **w**_*i*_ that are neighboring at an early stage of the adaptation procedure might not be neighboring anymore at a more advanced stage.

#### 2.4.2. Minimal Free Energy Vector Quantization

Another important clustering method represents the minimal free energy vector quantization [14, 15]. It is a divisive procedure starting first with one cluster containing the whole data set. During the learning process, the number of clusters increases and their size decreases as they specialize on specific data distributions. Other than fuzzy *c*-means clustering, this algorithm does not operate with prespecified cluster centers [13, 16].

The adaptation paradigm for this technique is [14]

(3) $$w_{i}\left(t+1\right)=w_{i}\left(t\right)+\epsilon \left(t\right)\frac{\exp\;-{||x\left(t\right)-w_{i}\left(t\right)||}^{2}/2\rho^{2}}{\sum_{i}\exp\;-{||x\left(t\right)-w_{i}\left(t\right)||}^{2}/2\rho^{2}}\left(x\left(t\right)-w_{i}\left(t\right)\right),$$

where *ρ* is the “fuzzy range” of the model and defines a length scale in data space and is annealed to repeatedly smaller values in the VQ approach.

The cooperativity function *a*_*i*_ = (exp −||**x**(*t*) − **w**_*i*_(*t*)||^2^/2*ρ* ^2^)/(∑_*i*_exp −||**x**(*t*) − **w**_*i*_(*t*)||^2^/2*ρ* ^2^) represents the so-called* softmax* activation function, and accordingly the outputs lie in the interval [0,1] and they sum up to one.

The main advantages of fuzzy clustering based on deterministic annealing over SOM were pointed out in [15]: (1) the divisive and multiresolution aspect of data analysis, (2) different control parameters (free energy, entropy) control and enable a direct cluster splitting, and (3) the gradient descent type of the learning rule monitored by an explicitly given error function.

### 2.5. Lesion Segmentation Methods

In the following, we will present the two employed segmentation methods for the evaluation of signal intensity time courses for the differential diagnosis of enhancing lesions in breast MRI.

#### 2.5.1. Conventional Segmentation Method

The clinical standard method to analyze dynamic MRI of the breast is based on carefully choosing a region of interest (ROI) surrounding the contrast enhancing lesion [10]. Lesion segmentation was performed automatically by a region growing algorithm after positioning a seed within the lesion. For all the voxels belonging to this ROI, an average signal intensity (SI) time curve was computed. In the present study, we selected only lesions with an initial contrast enhancement ≥50% for comparative analysis between the standard evaluation method and unsupervised clustering. Thus, we use a semiautomatic segmentation method to determine the ROI including all voxels of a lesion with an initial contrast enhancement of ≥50%.

The initial contrast enhancement was calculated according to the following equation:

(4) $$\begin{array}{c} sa_{i}=\frac{\left(\text{S}{\text{I}}_{\text{2nd frame post-contrast}}-\text{S}{\text{I}}_{\text{pre-contrast}}\right)}{\text{S}{\text{I}}_{\text{pre-contrast}}}\times 100\text{\%} \end{array}.$$

The postinitial signal course was calculated based on the following equation:

(5) $$sv_{p}=\frac{\left(\text{S}{\text{I}}_{5\text{th}\;\text{frame}\;\text{post-contrast}}-\text{S}{\text{I}}_{\text{maximum}\;1\text{st}\;\text{to}\;2\text{nd}\;\text{frame}\;\text{post-contrast}}\right)}{\text{S}{\text{I}}_{\text{maximum}\;1\text{st}\;\text{to}\;2\text{nd}\;\text{frame}\;\text{post-contrast}}}\times 100\text{\%}.$$

**Figure 3:**
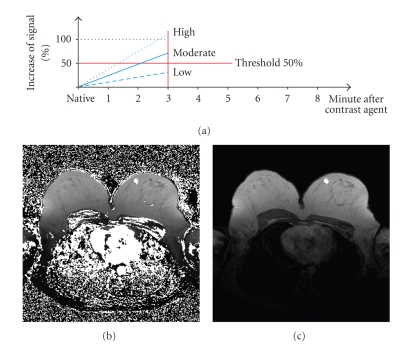
Conventional segmentation method. (a) Threshold segmentation. (b) Classification based on threshold segmentation: pixels exhibiting time signal intensity curves above a given threshold are white. (c) The lesion is determined based on region growing.

For all voxels belonging to this ROI an average time-signal intensity curve is computed for both initial signal increase and postinitial signal course. This averaged value is then rated. This very simply method is fast but is threshold-limited. Figure 3 illustrates the described segmentation method. White pixels exhibit an above-threshold signal increase. The contrast-enhanced pixels are shown in Figure 3(b). Based on a region-growing method [17], we can easily determine the suspicious lesion area.

#### 2.5.2. Segmentation Method Based on Unsupervised Clustering

This method uses the ROI of the previous segmentation method while all the voxels within this ROI are subject to cluster analysis. This allows the clinical radiologist to be a more detailed view of the signal curves by partitioning the ROI. This segmentation method reveals regional properties of contrast-agent uptake characterized by subtle differences of signal amplitude and dynamics as it is demonstrated by our results in Figures 6, 7, and 8. As a result, we obtain both a set of prototypical time-series and a corresponding set of cluster assignment maps which further provides a segmentation with regard to identification and regional subclassification of pathological breast tissue lesions.

For every single cluster a mean percentage signal intensity change- (PSIC-) curve is computed which contains only the signal information of the signal time curves that are assigned to this particular cluster. For example, if we perform clustering for *N* = 4 clusters, then we obtain 4 cluster-specific PSIC-curves. The obtained time-signal intensity curves of enhancing lesions were plotted and presented to two experienced radiologists who were blinded to any clinical or mammographic information of the patients. The radiologists were asked to rate the time courses as having a steady, plateau, or washout shape—types I, II, or III, respectively [2]. The cluster vector of each lesion with the highest classification level was used for further statistical evaluation.

## 3. Results

All lesions (*n* = 40) with an initial signal increase ≥50% after contrast injection were included in the comparative analysis of the conventional segmentation method and cluster analysis. Histological findings were malignant in 31 and benign in 9 lesions. Lesion size was determined as the number of voxels with an initial SI increase of ≥50%.

Clustering results were evaluated by (i) qualitative visual inspection of cluster assignment maps, that is, cluster membership maps according to a minimal distance criterion in the metric of the pixel-time course (PTC) feature space shown for the “neural-gas” network, (ii) qualitative visual inspection of corresponding cluster-specific time-signal intensity curves for the “neural-gas” network, (iii) optimal parameter combination, (iv) optimal number of clusters, (v) comparison between “neural-gas” network, minimal free energy vector quantization and conventional segmentation method, and (vi) receiver operating characteristic (ROC) analysis.

### 3.1. Parameter Optimization of the Unsupervised Classifier

The unsupervised classifier based on the “neural-gas” network, as shown in (2), has three free parameters: the initial step size *α*, the number of epochs being equal to the number of the training steps multiplied by the number of training data vectors, and the initial decay constant *λ*. We analyzed the whole possible range of parameters, corresponding thus to the largest range: *α* was varied between 0.1 and 1, the number of epochs and the initial decay constant were also varied according to [12]. We calculated the variations of parameters in terms of the initial and postinitial contrast enhancement according to (4) and (5) as these parameters are crucial for the correct detection. We tested 30 different parameter combinations with: (a) *α* being varied between 0.1 and 1, (b) number of epochs between 1 and 10, and (c) *λ* between *N*/6 and *N* with *N* being the number of clusters and additionally, *λ* = 3*N*/2,4*N*/2,5*N*/2,6*N*/2. The optimal combination of these three parameters is important to achieve stable clustering results, in the sense that the same clustering results are obtained regardless the initialization.

Our simulation results showed that an optimal parameter combination for small lesion detection is characterized by a small standard deviation of the minimum and maximum values of the percentage of the initial signal increase *s* *a*_*i*_ and the percentage of the postinitial signal intensity *s* *v*_*p*_.

**Figure 4:**
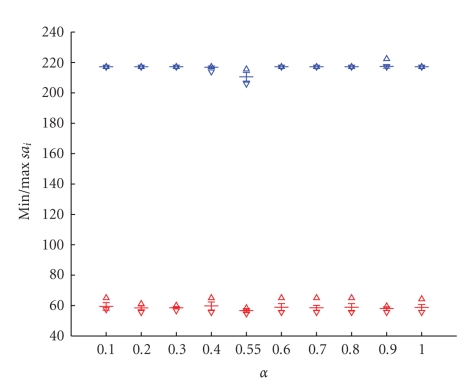
Variance of the minimal and maximal percentage of the initial signal increase *s* *a*_*i*_ for 20 runs in function of the initial steps size *α* varied from 0.1 to 1. As an optimal value, we obtain *α* = 0.3.

**Figure 5:**
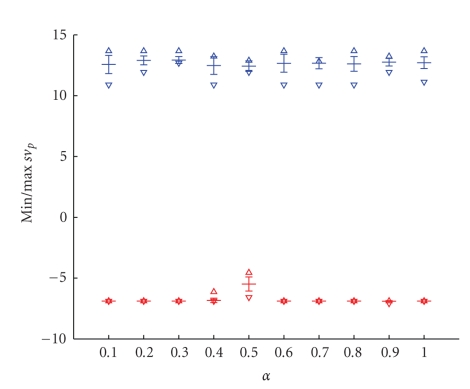
Variance of the minimal and maximal percentage of the postinitial signal intensity *s* *v*_*p*_ for 20 runs in function of the initial steps size *α* varied from 0.1 to 1. As an optimal value, we obtain *α* = 0.3.

Exemplary, we show in Figures 4 and 5 the minimal and maximal values for *s* *a*_*i*_ and *s* *v*_*p*_ for the variation of the initial step size *α*. Small variances indicate a stable clustering result. As an optimal value, we obtain *α* = 0.3.

The optimal parameter combination achieved for data set #1 is shown in Table 1.

### 3.2. Optimal Number of Clusters

An important aspect is to define the number of CVs prior to clustering. The exact number of clusters is usually determined by cluster validity techniques. In general, the higher the number, the finer grained the analyzed ROI is partitioned, however at the expense of an increase in signal noise susceptibility, while a lower number leads to overlooking of pertinent information. In [8], the number of clusters is determined as the number of voxels in the 3D breast lesion is divided by 80. In our study, we have experimented with different cluster numbers ranging from 2 to 8.

The distribution of different cluster numbers to the four Kuhl classes is shown in Table 2. The mean and standard deviation of the clusters' assignments to the Kuhl classes is represented in function of the cluster number. The results are for 20 runs performed on the actual kinetic curves using the same parameters for the neural network but with different codebook initializations and can be interpreted as follows: the smaller the value of the standard deviation, the better the reproducibility and the higher the significance of the simulation experiments. It becomes quickly evident that the optimal representation is achieved for four clusters: a zero standard deviation means a correct identification of the time-signal intensity curve types.

**Figure 6:**
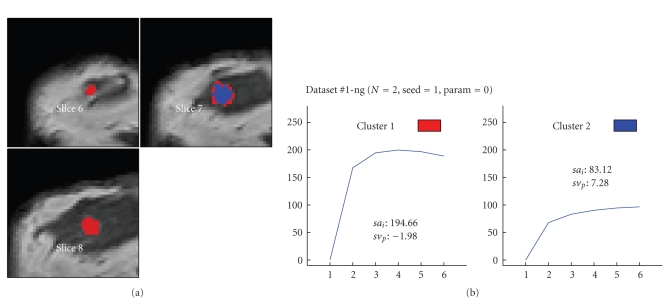
Segmentation based on the “neural-gas” network applied to data set #1 (malignant lesion, ductal carcinoma in situ (DCIS)) and resulting in two clusters. (a) shows the cluster distribution for each slice ranging from 6 to 8. (b) visualizes the representative time-signal intensity time curves for each cluster.

**Figure 7:**
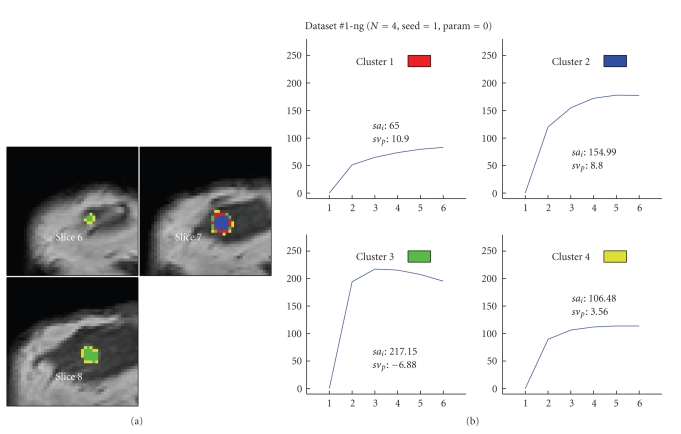
Segmentation method based on the “neural-gas” network applied to data set #1 (malignant lesion, DCIS) and resulting in four clusters. (a) shows the cluster distribution for each slice ranging from 6 to 8. (b) visualizes the representative time-signal intensity time curves for each cluster.

**Figure 8:**
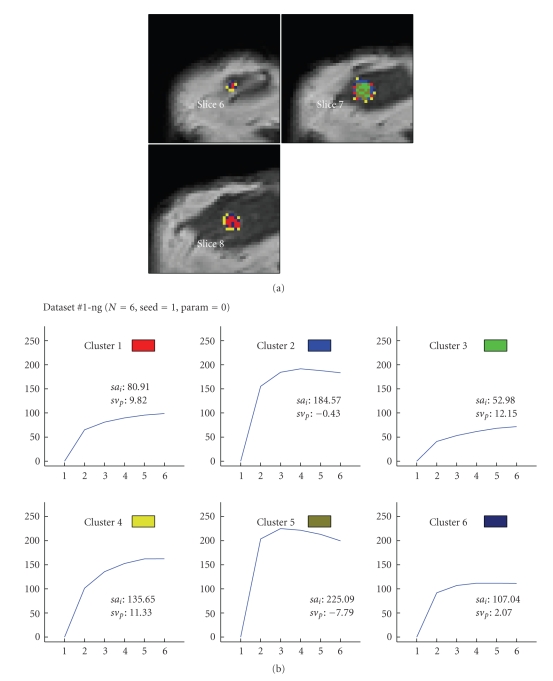
Segmentation method based on the “neural-gas” network applied to data set #1 (malignant lesion, DCIS) and resulting in six clusters. (a) shows the cluster distribution for each slice ranging from 6 to 8. (b) visualizes the representative time-signal intensity time curves for each cluster.

Figures 6 to 8 visualize the cluster distribution and the representative time-signal intensity time curves for two, four, and six clusters for data set no. 1.

All lesions with an initial signal increase ≥50% after contrast injection were included in the comparative analysis of the conventional segmentation method and the two cluster analysis methods. Histological findings were malignant in 31 and benign in 9 lesions. In this study, lesion segmentation was semiautomatically performed.

The results of the conventional segmentation method compared with the two unsupervised clustering methods were correlated with the histological findings and represented in Tables 3 and 4. The tables show the results for the 40 data sets. Senstitivity increased with unsupervised clustering in DCIS, in invasive ductal, and especially in lobular carcinoma while specificity remained constant.

Clustering is done for 20 different runs using the same parameters but different algorithms' initializations. The tables show an increased sensitivity of segmentation method based on both vector quantization methods compared to the conventional segmentation analysis in DCIS, lobular and invasive ductal carcinoma in comparison to the mean signal intensity time curve of the undivided ROI. “Neural-gas” shows an increase in sensitivity versus MFE [10] for the two latter carcinoma. Specificity remained constant for all benign lesions for the “neural-gas”, however decreased based on MFE for the benign lymph node. In summary, the use of clustering allows a more differentiated view of the hidden structure of the analyzed dataset. This method reveals information, else not evident. On the other hand, the “single” cluster of the undivided ROI contains too many information details. In summary, clustering can be a useful tool to extract this information from the signal.

### 3.3. ROC-Analysis

Clustering results were also evaluated by an ROC analysis. In the ROC curve, we compare both segmentation methods with the gold standard (histology). For both segmentation methods, we obtain for each of the 40 small lesions a value between 1 and 4 showing to each of the four curves from Figure 1 it belongs. For the benign lesions we have the following: type Ia has value 1 and type Ib has value 2. Similarly, for malignant lesions, we obtain the following: type II has value 3 and type III has value 4. To determine the ROC curves, we have to vary the threshold based on which a lesion is considered malignant. Each ROC curve has 6 parameters: 4 from the different classes and 2 corresponding to sensitivity = 1, specificity = 0, and sensitivity = 0, specificity = 1.

From Table 5, we see that the second segmentation method yields better results than the first one: in other words, the unsupervised classification outperforms the conventional segmentation method based on averaged above-threshold classification.

## 4. Conclusion

The goal of the presented study was the introduction of new approaches for the evaluation of dynamic MR mammography in * small lesions* and is motivated by the conceptual weaknesses of the conventional technique. We presented two different segmentation methods for the evaluation of signal intensity time courses for the differential diagnosis of small enhancing lesions in breast MRI. It is important to mention that the interpretation of kinetic curves is not standardized in the current clinical practice.

 A manually predefined ROI is substantially impacting the differential diagnosis in breast MRI by being both time-consuming and significantly suffering from inter- and intraobserver variability. On the other hand, cluster analysis is almost independent of manual intervention but is computationally intensive. The conventional method based on threshold segmentation allows a differentiation between contrast-enhanced lesions and surrounding tissue. However, a subdifferentiation within the lesion is not provided. A fusion of the techniques of threshold segmentation and cluster analysis combines the advantages of these single methods. Thus, a fast segmentation method is obtained which carefully discriminates between regions with different lesion enhancement kinetics. The average computational time needed for evaluation was of two seconds. We have determined the optimal number of clusters and thus the optimal number of characteristic curves for lesion segmentation and classification. A small number will lead to a characteristic curve being very close to the average curve, and thus not able to subdifferentiate the lesion. A larger number, however, makes the characteristic curve prone to noise sensitivity. The performed ROC-analysis shows that the unsupervised clustering technique represents a valuable tool for supporting radiological diagnosis in dynamic breast MR imaging. However, the most important advantage lies in the potential of increasing the diagnostic accuracy of MRI mammography by improving the sensitivity without reduction of specificity for the data sets examined.

## Figures and Tables

**Table 1:** Optimal parameter combination for the “neural-gas” network optimized for data set #1 when choosing *N* = 4 clusters.

Parameter	Step size *α*	Nr. epochs	Decay constant *λ*
	0.3	10	*N*/4

**Table 2:** Distribution of the four classes from Figure 1 for different number of clusters, *N* = 2 to 8, for 20 different runs. Represented are in function of the number of clusters the mean and standard deviation of the clusters to the four Kuhl classes Ia, Ib, II, and III.

Number	Standard deviation				Mean			
	Ia	Ib	II	III	Ia	Ib	II	III
2	0	0	0	0	1	0	1	0
3	0	0	0.513	0.513	1	1	0.5	0.5
4	0	0	0	0	1	2	0	1
5	0.366	0.447	0.324	0.224	1.15	1.9	1	0.95
6	0.51	0.51	0.5	0.5	1.55	1.45	1.6	1.4
7	0.224	0.51	0.51	0.224	1.95	1.55	1.55	1.95
8	0.696	0.671	0.6	0	2.2	2.35	1.45	2

**Table 3:** Comparison of the conventional segmentation method and minimal free energy vector quantization and “neural-gas” network in the detection of malignant lesions.

Histology	Number	Conventional segmentation method	MFE	Neural Gas
		True positive	True positive	True positive
DCIS	5	60%	80%	80%
Invasive ductal	17	82%	88%	94%
Invasive lobular	4	75%	75%	100%
Scirrous carcinoma	3	66%	66%	66%
Medullary carcinoma	1	100%	100%	100%

**Table 4:** Comparison of the conventional segmentation method and minimal free energy vector quantization and “neural-gas” network in the detection of benign lesions.

Histology	Number	Conventional segmentation method	MFE	Neural Gas
		True negative	True negative	True negative
Mastopathy	3	33%	33%	33%
Fibroadenoma	3	66%	66%	66%
Granuloma	1	100%	100%	100%
Benign lymph node	1	100%	0%	100%
Scar, no relapse	1	100%	100%	100%
Papilloma	1	0%	0%	0%

**Table 5:** Results of the comparison between the two different segmentation methods for the 40 small lesions: the average area under the curve *A*_*Z*_ and its deviations for 20 different ROC runs using the same parameters but different algorithms' initializations.

Segmentation method	Area under the curve *A*_*Z*_
Conventional segmentation analysis	0.6207
“Neural-gas” network	0.8388
